# Heart Failure Risk Predictions and Prognostic Factors in Adults With Congenital Heart Diseases

**DOI:** 10.3389/fcvm.2022.692815

**Published:** 2022-02-24

**Authors:** Patryk Leczycki, Maciej Banach, Marek Maciejewski, Agata Bielecka-Dabrowa

**Affiliations:** ^1^Department of Cardiology and Congenital Diseases of Adults, Polish Mother's Memorial Hospital Research Institute, Łodź, Poland; ^2^Department of Preventive Cardiology and Lipidology, Medical University of Lodz, Łodź, Poland

**Keywords:** prognostic factor, adults with congenital heart disease (ACHD), congenital heart defect (CHD), risk prediction, heart failure, congenital heart disease

## Abstract

In recent decades the number of adults with congenital heart diseases (ACHD) has increased significantly. This entails the need for scrupulous evaluation of the current condition of these patients. The ACHD population is one of the most challenging in contemporary medicine, especially as well-known scales such as the NYHA classification have very limited application. At the moment, there is a lack of universal parameters or scales on the basis of which we can easily capture the moment of deterioration of our ACHD patients' condition. Hence it is crucial to identify factors that are widely available, cheap and easy to use. There are studies showing more and more potential prognostic factors that may be of use in clinical practice: thorough assessment with echocardiography and magnetic resonance imaging (e.g., anatomy, ventricular function, longitudinal strain, shunt lesions, valvular defects, pericardial effusion, and pulmonary hypertension), cardiopulmonary exercise testing (e.g., peak oxygen uptake, ventilatory efficiency, chronotropic incompetence, and saturation) and biomarkers (e.g., N-terminal pro-brain type natriuretic peptide, growth-differentiation factor 15, high-sensitivity troponin T, red cell distribution width, galectin-3, angiopoietin-2, asymmetrical dimethylarginine, and high-sensitivity C-reactive protein). Some of them are very promising, but more research is needed to create a specific panel on the basis of which we will be able to assess patients with specific congenital heart diseases.

## Introduction

In recent decades the number of adults with congenital heart diseases (ACHD) has increased significantly, mostly due to substantial progress in pediatric cardiac surgery and cardiology (1). Accurate prevalence of ACHD is not known and difficult to estimate. Nevertheless, the prevalence of ACHD is rapidly growing, exceeding the prevalence of congenital heart defects in the pediatric population two-fold (1).

More and more adult patients with CHD present advanced forms of heart failure (about 20–50% of this population) which is main cause of death in ACHD population, and their quantity will be growing (2–4). Patients at high risk of developing heart failure require systematic follow-up and diagnostic screening. The knowledge we have about diagnosis and treatment of HF has very limited usage in ACHD. First of all, unlike in the general population, adults with CHD form a very heterogenous population what impedes the creation of uniform standards of treatment. Prevalence of signs and symptoms of HF seems to be the highest in patients with functionally univentricular hearts post-Fontan palliation (40%), in congenitally corrected TGA patients (32%) and in patients with complete TGA (22%) (5). The pathophysiology of HF in ACHD, unlike in the general population, is much more complex. It includes multiple causes: ventricular dysfunction caused by pressure/volume overload and compromised systolic ventricular function due to incorrect myocardial architecture, myocardial injuries during numerous surgeries and invasive interventions, ischemic heart disease that is based on congenital coronary arteries anomalies and arrhythmias (6). Secondly, there is very slow progress in the management of HF in CHDs, and many modern therapies has no proven benefits in this group of patients. Thirdly, we do not have an universal tool with which we can easily determine the severity of impaired physical functionality in ACHD (functional class)—the NYHA classification has limited usage due to overall tolerance of limited functionality in patients with CHDs. CHD present from birth, and patients adapt daily activities to their ability, thus, underestimating and under-reporting the severity of physical limitation (7).

The purpose of this work is to review current prognostic factors in ACHD and their significance in the diagnosis of heart failure, prognosis and management of patients with CHD and accompanying HF.

## Materials and Methods

We searched the electronic database PUBMED (2000–2021). Additionally, data from international cardiovascular meetings and guidelines of cardiovascular societies were searched. The main data search terms were: adult congenital heart disease, echocardiography, magnetic resonance imaging, exercise, cardiopulmonary exercise testing, biomarker(s), marker(s), heart failure, NYHA class, prognosis, risk factor(s).

### Prognostic Factors in Patients With CHD

#### Transthoracic Echocardiography in Assessment of Prognostic Factors in ACHD

The most basic parameter obtained in TTE is the systolic ventricular function (as expressed by an ejection fraction with the biplane Simpson's method), which is a well-established prognostic factor. Koyak et al. showed that moderately to severely impaired systemic ventricular function and moderate to severe subpulmonary ventricular dysfunction are predictors for sudden cardiac death (SCD) in the general CHD population (8). In adults with repaired tetralogy of Fallot, the link between moderate or severe left ventricular systolic dysfunction and very wide QRS complex (≥180 ms) had positive (66%) and negative (93%) predictive value for SCD (9). Baggen et al. reported that size and function of the left atrium (left atrial volume index ≥34 mL/m^2^, total and active emptying fraction but especially length) were significantly associated with cardiovascular events (10). The authors concluded also that a normally sized left atrium could accurately rule out the risk of death and heart failure with a high negative predictive value (10). Also, it was reported that left ventricular longitudinal dysfunction assessed with strain was associated with SCD and life-threatening arrhythmias (11) and tricuspid or pulmonary valve regurgitation was a prognosticator of death (12). In post-fontan procedure patients the systolic to diastolic duration ratio has been described as one of the mortality predictors (systolic duration was measured from the onset to the end of AV valve regurgitation; diastolic duration was measured from the end of AV valve regurgitation to the onset of the subsequent AV valve regurgitation signal) (13). Additionally, in patients with a systemic right ventricle it seems that longitudinal systolic strain is related to elevated NT-proBNP, progression in New York Heart Association class, relevant arrhythmia and death (14, 15). In the Eisenmenger population tricuspid annular plane systolic excursion (TAPSE <15 mm) and ratio of right ventricular effective systolic to diastolic duration (≥1.5; systolic duration was measured from the onset to the end of tricuspid regurgitation and diastolic duration from the end of tricuspid regurgitation to the onset of the subsequent tricuspid regurgitation signal) have been of predictive value (16, 17). Moceri et al. proposed echocardiographic score based on the strongest echocardiographic predictors of outcome, including 1 point for each of the following: tricuspid annular plane systolic excursion <15 mm, ratio of right ventricular effective systolic to diastolic duration ≥1.5, RA area ≥25 cm^2^, ratio of RA to left atrial area ≥1.5, that was highly predictive of mortality (16). Therefore, one may consider to incorporate it routinely in the assessment of Eisenmenger patients. Abnormal right atrial area (≥25 cm^2^) and ratio of right atrial to left atrial area (≥1.5) have also been reported as a bad prognostic factors in patients with Eisenmenger syndrome and pulmonary hypertension (16, 17). Additionally, pericardial effusion or pretricuspid shunt were reported to be a prognosticator of death (18).

#### Magnetic Resonance Imaging and Cardiovascular Computed Tomography in Assessment of Prognostic Factors in ACHD

Ventricular size and function obtained by MRI are well-known prognostic factors of adverse outcomes such as mortality and other adverse events in many groups of patients with congenital heart diseases. Especially accurate quantification of right ventricle size and function and pulmonary regurgitation is particularly important in patients with tetralogy of Fallot„ transposition of the great arteries (TGA) and systemic right ventricle (14, 19–23). Ortega et al. compared data from cardiac magnetic resonance (CMR) between patients with repaired ToF with (*n* = 13) and a control group without (*n* = 26) histories of death and sustained ventricular tachycardia (VT), finding that left ventricle synchrony measured with CMR tissue tracking is associated with death and VT (20). Rathod et al. on the other hand retrospectively reviewed CMR data of patients with Fontan circulation (*n* = 215), revealing that increased ventricular indexed end-diastolic volumes (>125 mL/body surface area) are not risk factors of elevated mortality or transplant (24). Van der Bom et al. published data showing that right ventricular end-diastolic volume index higher than 150 ml/m^2^ is associated with increased risk of adverse events in patients with a systemic right ventricle (25). Impaired longitudinal strain of left and right ventricle measured by MRI also seems to be closely related to higher risk of adverse events (26). In a population of post-fontan operation patients, end diastolic volume index, end systolic volume index, contractility, mass index and increased afterload were identified as independent factors of higher morbidity and mortality (27). A recent study showed that stroke volume (SV) ratio (<0.95; SV of the two whole ventricles/SV of the right ventricle + SV of the left ventricle), aorta arising from the right ventricle conus, biventricular ejection fraction (<0.45), and left ventricular indexed end-diastolic volume (>73 mL/m^2^) calculated using MRI can be used as predictive factors of cardiac events in patients with biventricular heart after Fontan surgery (28). Results of single studies suggest that the deformation imaging technique in MRI and myocardial fibrosis assessed by late gadolinium enhancement and T1 mapping may be very helpful prognostic tools in the future (20, 29–34). Increased late gadolinum enhancement of the left ventricle in patients with repaired ToF is related to myocardial dysfunction and is associated with adverse outcomes after correcting for age (34). Additionally, in patients with ToF and pulmonary arterial hypertension its location at the right ventricular insertion points is thought to reflect a more advanced disease and poor prognosis (29). Correlation between septal interstitial expansion representing diffuse fibrosis with NT-proBNP levels and chronotropic index in patients with a systemic right ventricle after correction of TGA seems to play role in the development of RV systolic impairment (31).

Cardiovascular computed tomography (CCT) is particularly useful in dissection, pulmonary embolism, and paravalvular abscess in the setting of endocarditis, where it may have advantages over echocardiography and CMR due to being less susceptible to prosthetic valve artifact. It is useful in assessment of coronary artery pathology and collaterals (35) (Table 1).

**Table 1:** Potential heart failure prognostic factors in patients with congenital heart disease.

	**Parameters**	**References**
Transthoracic echocardiography	– Ventricular and atrial anatomy – Systolic ventricular function – Systolic to diastolic duration ratio – Shunt lesions – Valvular defects – Pericardial effusion – LS	(3, 8, 10, 16, 17) (3, 9, 11) (13) (3, 18) (3, 12) (18) (14, 15)
Magnetic resonance imaging	– Anatomy – Ventricular function (R/L VEF, R/L VEDV, R/L VEDV index) – Mass index – R/L LS – SV ratio – Myocardial scarring and fibrosis – T1 mapping	(3, 22, 23) (3, 19, 22, 23, 25, 27, 28) (3, 23, 27) (3, 26) (28) (3, 29, 31, 34) (3, 30)
Cardiopulmonary exercise testing	– VO_2_peak – VE/VCO_2_ slope – Peak HR – HRR – HRr – W – Maximal sBP – Saturation – Mean peak RER – Borg scale – OUES – VO_2_/WR – VAT	(3, 7, 36–43) (3, 36, 39, 44, 45) (3, 36, 38, 42, 46–49) (3, 36, 38, 42, 46–49) (3, 36, 38, 42, 46–49) (51) (25) (3, 36, 50) (51) (51) (51) (51) (51)
Biomarkers	– NT-proBNP – GDF-15 – hs-TnT – RDW – Galectin-3 – Ang-2 – ADMA – hsCRP	(3, 6, 55–59) (57, 60–63) (3, 57, 64) (65, 66) (67) (68) (69) (3, 70)

*LS, longitudinal systolic strain; R/L, right/left; VEF, ventricular ejection fraction; VEDV, ventricular end diastolic volume; SV, stroke volume; VO_2_, oxygen uptake; VE/VCO_2_, ventilatory efficiency; HR, heart rate; HRR, heart rate reserve; HRr, heart rate recovery; W, peak load; sBP, systolic blood pressure; RER, respiratory exchange ratio; OUES, oxygen uptake efficiency slope; VO_2_/WR, oxygen uptake to work rate; VAT, ventilatory anaerobic threshold; NT-proBNP, N-terminal pro-brain type natriuretic peptide; GDF-15, growth-differentiation factor 15; hs-TnT, high-sensitivity troponin T; RDW, red cell distribution width; Ang-2, angiopoietion-2; ADMA, asymmetrical dimethylarginine; hsCRP, high-sensitivity C-reactive protein*.

#### Cardiopulmonary Exercise Testing (CPET, CPX) in Assessment of Prognostic Factors in ACHD

CPET, including assessment of objective exercise capacity (peak oxygen consumption peak VO_2_), ventilatory efficiency [ventilation to carbon dioxide output (VE/VCO_2_) slope], chronotropic and blood pressure response, as well as exercise-induced arrhythmia and desaturation, improve the assessment of functional capacity and physical fitness and correlate well with morbidity and mortality in ACHD patients (7). The peak VO_2_ is one of the most useful and potent prognostic factor due to its strong association with increased risk of heart failure, hospitalization and mortality in ACHD (7, 36–39). According to data presented by Diller et al. peak VO_2_ <15.5ml•kg^−1^•min^−1^ was associated with the greatest risk of hospitalization or death and death alone in adults with congenital heart diseases (7). In the population of patients with Ebstein anomaly peak VO_2_ relates to severity of the underlying disease (38). VO_2_ ≤ 52.3% of predicted value was associated with higher 4-year risk of death or cardiac related emergency hospital admission in patients after a Mustard and Senning operation (39). In the whole population of CHD patients peak VO_2_ is reduced (37, 40, 41) to differing degrees depending on the type of heart defect (37, 41, 42). Kempny et al. published data showing that peak VO_2_ was lowest in the Eisenmenger population and complex lesions including univentricular hearts and patients with cyanosis (mean ± SD: 43 ± 13 and 46 ± 16% of predicted peak VO_2_, respectively) and highest in TGA patients after an arterial switch operation, patients with various forms of valvular heart disease and repaired aortic coarctation (mean ± SD: 89 ± 22, 80 ± 30, and 78 ± 25% of predicted peak VO_2_, respectively) (41). Diller et al. reported that peak VO_2_ was normal in only 1% and borderline in 2.7% of patients with Fontan circulation (42). Recently published data have demonstrated a strong inverse relation of NYHA class with VO_2_ peak and additionally with oxygen uptake efficiency slope (OUES) and double product at peak exercise (product of heart rate and systolic blood pressure). However, there was also high inconstancy in exercise capacity measured in various NYHA classes (43). Dimopoulos et al. published data showing that CHD patients presented higher VE/VCO_2_ slope than healthy individuals and simultaneously revealed VE/VCO_2_ slope as the most potent predictor of mortality in non-cyanotic patients (2-year survival of 86% with VE/VCO_2_ slope >38 vs. 99% with VE/VCO_2_ slope <38) (44), which was also presented in a study by Inuzuka et al. (36). In cyanotic ACHD VE/VCO_2_ slope did not have predictive ability (36, 44). Predictive value of VE/VCO_2_ slope has been confirmed in the population of repaired ToF patients (VE/VCO_2_ slope ≥ 31 alongside peak VO_2_% ≤ 65% and QRS duration ≥170 ms were associated with the best sensitivity and specificity to predict adverse outcome) (45) and TGA patients after a Mustard and Senning operation (VE/VCO_2_ ≥35.4 and VO_2_% ≤ 52.3% were associated with an increased 4-year risk of death/cardiac-related emergency hospital admission; 4-year event rate of 78.8%) (39). Also, heart rate response in the ACHD population may be abnormal (36, 46–48). Diller et al. in a population of 727 ACHD patients found abnormal heart rate response to be associated with greater risk of death and to affect 62% of the population—the lowest prevalence was observed in patients with ventricular septal defect, Ebstein anomaly, transposition of the great arteries and the highest in patients with complex, cyanotic and uncorrected defects (46). Reduced values of such parameters as peak heart rate, heart rate reserve (HRR), and heart rate recovery have been introduced as predictors of mortality (38, 42, 46, 49). Especially, abnormal heart rate reserve puts ACHD patients in a higher risk group. What is interesting, in patients with Eisenmenger syndrome neither HRR nor VO_2_ peak was able to predict prognosis (46). Radojevic et al. published data showing that heart rate reserve <25 beats per minute was of predictive value in patients with Ebstein anomaly (38) and in the analysis of Diller et al. HRR (with a cut-off value of 72 beats per minute) was suggested to be the only significant prognostic marker for mortality or transplantation in contrast to any other parameter of CPET in the population of Fontan patients (42). A study involving 345 ACHD patients showed that chronotropic incompetence (defined as the inability to achieve 80% of the predicted maximal heart rate) placed patients in a higher NYHA class and was related to higher NT-proBNP levels and lower VO_2_ peak (49). Single studies point out other measurements in specific CHD groups that may be important: peak systolic blood pressure during uppermost exercise lower than 180 mmHg combined with a right ventricular end-diastolic volume index above 150 mL/m^2^ in patients with a systemic right ventricle (25), >5% decrease in saturation during exercise or to <90% (36, 50). Buys et al. compared ACHD (coarctation of the aorta, ToF, dextro-TGA, and univentricular heart) with healthy adults and found mean peak respiratory exchange ratio (RER), median Borg scale, oxygen uptake efficiency slope (OUES), oxygen uptake (VO_2_) to work rate (WR) relationship and ventilatory anaerobic threshold (VAT) to be notably reduced in the CHD population (lowest in univentricular heart population) (51). This leaves an open field for further investigations of these parameters and many others for which we could not find any studies that assessed their statistical significance in risk stratification. Serial exercise testing might be a part of long-term follow-up protocols in ACHD patients. It plays an important role in the timing of interventions and reinterventions (3).

#### Six Minutes Walk Test in Assessment of Exercise Tolerance in ACHD

**Figure 1:**
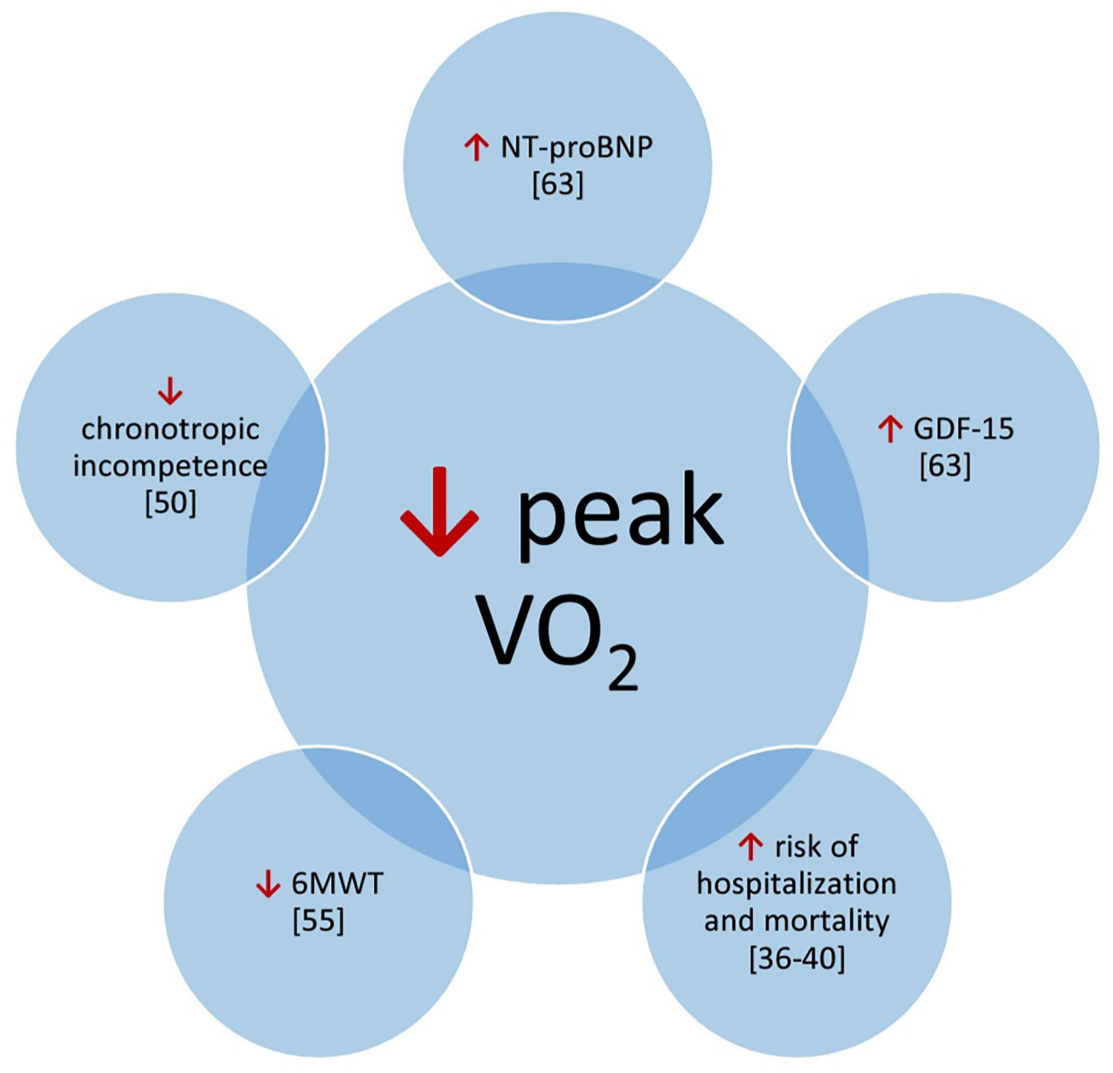
Potential correlations of prognostic factors with peak V02. 6MWT, 6 minute walk test; GDF-15, growth-differentiation factor 15; NT-proBNP, N-terminal pro-bra in type natriuretic peptide.

The 6-min walk test (6MWT) is another simple test for quantification of exercise capacity; it relates to outcome in patients with pulmonary arterial hypertension (2, 3). Kempny et al. showed that patients with Eisenmenger syndrome who had baseline saturation below 85% or did not reach 350 m in the 6-min walk test (6MWT) had 3 times greater risk of death (52). In Eisenmenger patients with reduced exercise capacity (6MWT distance <450 m), a treatment strategy with initial endothelin receptor antagonist monotherapy should be considered followed by combination therapy if patients fail to improve (3). The 6-min walk tests performed in patients with CHD have revealed a correlation with concentration of BNP. Additionally, there have been established a cut-off value (450 m) that allows one to categorize patients into groups of BNP concentration below or above 100 pg/ml (53). Another study concerning 6MWT established a cut-off value (482 m) of 6MWT distance that was able to select patients with reduced VO_2_peak (54). Moreover, multivariate regression analysis distinguished the 6MWT distance and NYHA classes as significant predictors of VO_2_peak (Figure 1) (54).

#### Biochemical Markers in ACHD

In CHD patients, natriuretic peptides [B-type natriuretic peptide (BNP) and N-terminal-pro-BNP (NT-pro-BNP)] are best studied and especially useful in patients with biventricular circulation and less useful in patients with a Fontan circulation (55). Eindhoven et al. published data showing that N-terminal pro-brain type natriuretic peptide (NT-proBNP) levels depend on diagnosis and are strongly associated with echocardiographic parameters and exercise capacity (56). Therefore, BNP and NT-proBNP are very important prognostic factors of mortality and may be a helpful tool for risk stratification and monitoring ACHD, as more and more other studies confirm (6, 57–59). In patients with heart failure, BNP/NT-proBNP were related to age, and BNP was more related to age than NT-proBNP. However, the influence of age on the levels of all natriuretic peptides was modest, and comparable to several other factors (71). Popelova et al. published data showing that the best discrimination value of NT-proBNP for mortality prediction in ACHD was 630 pg/ml (74 pmol/L; sensitivity 74%, specificity 84%) (58). Additionally, baseline measurements of NT-proBNP were closely related to increased risk of death (58, 59). Nevertheless, NT-proBNP level below 15.2 pmol/L can assuredly place patients in the low risk category of death or heart failure (57). In the study of Baggen et al. it was found that changes of NT-proBNP levels were associated with higher risk of adverse events. The annual increase of ~25% of the baseline value is suggested to be of clinical importance. Interestingly, some patients (25%) with the greatest decline in NT-proBNP levels also were at higher risk of adverse events (59). In the position paper of the working group of grown-up congenital heart disease and the heart failure association of the European Society of Cardiology, it was proposed that in ACHD a double increase in NT-proBNP level within 6 months should be considered as a significant rise (6). NT-proBNP should be the basic parameter assessed in the population of ACHD—elevated levels and, above all, increases in time are prognostically relevant in ACHD. Serial testing of natriuretic peptides plays a role in identifying patients at risk for adverse events. There are also other biomarkers that potentially can be used to predict adverse outcomes. Data presented by Baggen et al. have shown that patients with the highest levels of GDF-15 (growth differentiation factor 15) (fourth quartile; >867 ng/L) had remarkably higher risk of unfavorable events in comparison with the lowest levels (first quartile; <487 ng/L). They also presented data showing a correlation between NT-proBNP and GDF-15 (*r* = 0.50) (57). In the study of Norozi, levels of growth differentiation factor 15 (GDF-15) were compared within subgroups of VO_2_max (<65%, 65–85%, >85%) and NT-proBNP (<100, 100–300, >300 pg/mL). As a result, levels of GDF-15 corresponded with levels of NT-proBNP and values of VO_2_max—the highest mean GDF-15 was in groups of VO_2_max <65% and NT-proBNP >300 pg/mL, the lowest in groups of VO_2_max >85% and NT-proBNP <100 pg/mL (60). Eindhoven et al. published data showing its positive correlation with NT-proBNP (*r* = 0.445, *p* < 0.001). Additionally, level of GDF-15 higher than the reference value (1,109 ng/L) was associated with NYHA class and decreased exercise capacity (61). One study conducted in a child population also showed that plasma GDF-15 levels were positively correlated with concentration of NT-proBNP (*r* = 0.810; *p* < 0.01) and negatively with left ventricular ejection fraction (*r* = −0.391; *p* < 0.01) (62). Interestingly, GDF-15 was also higher in patients with elevated pulmonary pressure (median 1,114 ng/L) in relation to patients with normal pulmonary pressure (median 606 ng/L) (61) and was associated with thromboembolic events (57). This was confirmed in a child population in which Li et al. detected significantly elevated levels of GDF-15 in patients with pulmonary arterial hypertension secondary to CHD (median 1,415 vs. 890.6 ng/L; *p* < 0.01) (63). Thus, GDF-15 and its correlation with NT-proBNP and VO_2_ might be potentially a substitute marker for identifying CHD patients at risk of heart failure. Also, a correlation of high-sensitivity troponin T (hs-TnT) and NT-proBNP has been presented by Eindhoven et al. (64) (*r* = 0.400, *p* < 0.001) and Baggen et al. (57) (*r* = 0.42). Elevated levels of hs-TnT (>14 ng/L) has also been observed in patients with systemic systolic dysfunction, non-sinus rhythm, second and higher NYHA class and especially systemic right ventricle or elevated pulmonary pressures (64). Baggen et al. stated that we may use NT-proBNP as a conventional risk marker (especially with its high negative predictive value) but we may use GDF-15 and hs-TnT in order to further identify patients in the highest cardiovascular risk—a combination of elevated concentrations of NT-proBNP, GDF-15 (>1,109 ng/L) and hs-TnT (>14 ng/L) placed patients with CHD in a group of the highest risk of adverse events (57). Hs-TnT alongside GDF-15 also seems to correlate with NT-proBNP. Therefore, one should take into cosideration not only NT-proBNP but also GDF-15 and hs-TnT because by assessing all three parameters, one is more likely to select the highest-risk patients. There are more parameters that might be of use in the future. Two recent studies have shown that elevated red cell distribution width (RDW) is a significant and independent predictor of adverse events in ACHD and thus may be used as another marker in risk stratification of this population, and taking into account that this parameter is available easily and with minimal cost in all laboratories and ambulatories, it might be a first line screening tool (65, 66). Galectin-3 is another biomarker which might be useful in risk stratification of CHD patients. Baggen et al. published data showing its association with age, cardiac medication use, NYHA classification, loss of sinus rhythm, cardiac dysfunction, NT-proBNP and adverse cardiovascular events, but this association disappeared after adjustment for NT-proBNP (67). Angiopoietin-2 (Ang-2) is also a potential marker for heart failure in the CHD population. Lukasz et al. found that levels of Ang-2 reached a statistically significant difference in patients with normal ventricular function compared to a group with severe ventricular dysfunction (3.53 ± 4.19 ng/ml vs. 7.48 ± 7.57 ng/ml, *p* < 0.05) and patients in NYHA class I (2.52 ± 1.6 ng/ml) compared to NYHA class II (6.83 ± 6.56 ng/ml, *p* < 0.0001) as well as NYHA class III (8.23 ± 6.88 ng/ml, *p* < 0.0001). Ang-2 was also elevated in patients with limited cardiopulmonary exercise. Additionally, elevated concentrations of Ang-2 were observed in patients with single ventricle physiology (11.21 ± 6.94 ng/ml) compared to those with a systemic left (2.93 ± 2.75 ng/ml, *p* < 0.0001) or right ventricle (4.86 ± 5.22 ng/ml, *p* < 0.0001), unlike NT-proBNP. Ang-2 is a promising biomarker, especially in the population of Fontan patients (68). Ang-2 Tutarel et al. published data suggesting asymmetrical dimethylarginine (ADMA) to be remarkably elevated in the CHD population and to be superior to NT-proBNP in differentiation of NYHA class and at least as good for assessment of exercise capacity in the CHD population. There were significant differences in ADMA levels between NYHA class I (0.44 ± 0.06 mmol/l) and NYHA class II (0.48 ± 0.08 mmol/l, *p* = 0.04) and III (0.54 ± 0.10 mmol/l, *p* < 0.001), as well as between NYHA class II and III (*p* = 0.02). Differences in NT-proBNP were also significant but not between NYHA class II and III. (69). A single study conducted by Opotowsky et al. showed that high-sensitivity CRP (hsCRP) level in the highest quartile (≥2.98 mg/L) is associated with inferior functional status and exercise capacity and increased risk for death, and most of all, is an independent risk factor of adverse cardiovascular events and all-cause mortality. Additionally, excluding patients with very high levels of hsCRP (potential infection; ≥10.0 mg/L) and women using estrogen-containing medications from the analysis had no effect on the results (70).

#### Risk Prediction Scales in ACHD

Some investigators have already tried to develop or apply existing scales and models of adverse outcomes risk prediction and poor exercise capacity to the population of CHD patients (36, 72–75). Yap et al. identified risk factors (poor functional class, pulmonary hypertension, valvular heart disease, single-ventricle physiology) in adults with CHD and atrial arrhythmias and based on them created a risk score (1 point for each predictor; 0—low risk, 1—moderate risk, >1 high risk) (72). The Low Heart Failure Survival Score has been found to be correlated with adverse events but has not been successful in adequate risk stratification in the CHD population (73). The Seattle Heart Failure Model also seems to not fully represent actual survival of patients with CHD (74). Another study showed no significant differences between the ABILITY index (it scores and assigns patients on their capacity to work, capacity to be active, and ability to go through uncomplicated pregnancies—if applicable; it assigns patients to 4 classes) and NYHA classification in assessing the CHD population (75). Nevertheless, they can be used as a help in identifying patients who are at greater risk of adverse events, but more studies are needed in order to achieve more accurate prediction. Still, it is very important to find a parameter or group of parameters on the basis of which it would be possible to define in an objective and simple way the degree of functional limitation in ACHD like the New York Heart Association classification in heart failure patients.

In ACHD patients any haemodynamic abnormalities, including arrhythmias, potentially causing heart failure that can be addressed by intervention or surgery must be excluded. The structural interventions not pharmacological treatment are the therapy of choice in this group of patients. There is the need for personalized risk assessment in ACHD involving not only signs of heart failure and/or arrhythmia but also accurate diagnosis of residual defects and possibilities of their interventional treatment and typing the best time for intervention. No ideal risk stratification scheme has so far been developed. The future perspectives regarding ACHD risk assessment should take into account arrhythmias monitoring, serial cardiopulmonary exercise testing for objective assessment of exercise intolerance, the serial assessment of biochemical biomarkers and perhaps in the future also genetic may be additional reasonable tool. Echocardiographic and other imaging markers needs personalization depending of the type of congenital heart defect (Table 2).

**Table 2:** Congenital heart diseases and their prognostic factors in selected ACHD.

**CHD**	**Parameters**	**References**
Tetralogy of Fallot	– Ventricular function and anatomy – QRS complex width – Size and function of the left atrium – LS – Tricuspid/pulmonary valve regurgitation – Left ventricular dyssynchrony (MRI) – Increased late gadolinium enhancement of the left ventricle (MRI) – Peak VO_2_ – VE/VCO_2_ slope – Heart rate response – Oxygen saturation – NT-proBNP and another biochemical parameters	(3, 4, 8, 9) (9, 45) (3, 4, 10) (11) (12) (20) (34) (3, 4, 7, 36, 37, 41, 42) (3, 4, 7, 36, 37, 44, 45) (3, 4, 46) (3, 4) (3, 4, 6, 55–71)
Transposition of the great artieries	– Ventricular function and anatomy – Systemic right ventricular LS – Right ventricular end-diastolic volume index – Diffuse fibrosis (MRI) – Peak VO_2_ – VE/VCO_2_ slope – Heart rate response – Peak exercise systolic blood pressure – NT-proBNP and another biochemical parameters	(3, 4, 8) (14, 15, 26) (25) (31) (3, 4, 7, 36, 37, 39, 41, 42) (3, 4, 39, 44) (3, 4, 46) (25) (3, 4, 6, 55–71)
Post-fontan procedure	– Ventricular function and anatomy – Systolic to diastolic duration ratio – End diastolic and systolic volume index (MRI) – Contractility (MRI) – Mass index (MRI) – Increased afterload (MRI) – Stroke volume ratio in biventricular heart (MRI) – Aorta arising from the right ventricle conus in biventricular heart (MRI) – Biventricular ejection fraction (MRI) – Left ventricular indexed end-diastolic volume in biventricular heart (MRI) – Peak VO_2_ – VE/VCO_2_ slope – Heart rate response – Oxygen saturation – NT-proBNP and another biochemical parameters – Liver function	(3, 4, 8) (13) (27) (27) (27) (27) (28) (28) (28) (28) (3, 4, 7, 36, 37, 41, 42) (3, 4, 7, 36, 37, 44) (3, 4, 42, 46) (3, 4) (3, 4, 6, 55–71) (3, 4)
Ebstein anomaly	– Ventricular function and anatomy – Peak VO_2_ – VE/VCO_2_ slope – Heart rate response – Oxygen saturation – NT-proBNP and another biochemical parameters	(3, 4, 8) (3, 4, 7, 36–38, 41, 42) (3, 4, 7, 36, 37, 44) (3, 4, 38, 46) (3, 4) (3, 4, 6, 55–71)
Eisenmenger syndrome	– Ventricular function and anatomy – Right/left/biventricular function – TAPSE – Right ventricular effective systolic to diastolic duration ratio – Right atrial area – Right atrial to left atrial area – Pericardial effusion – Pretricuspid shunt – Oxygen saturation – Peak VO_2_ – 6-min walk test – NT-proBNP and another biochemical parameters	(3, 4, 8) (19) (3, 4, 16, 17) (16, 17) (16, 17) (16, 17) (18) (3, 4, 18) (3, 4, 19) (3, 4, 7, 36, 37, 41, 42) (52) (3, 4, 6, 55–71)

*LS, longitudinal systolic strain; VO_2_, oxygen uptake; VE/VCO_2_, ventilatory efficiency; NT-proBNP, N-terminal pro-brain type natriuretic peptide*.

## Conclusions

There is no possibility at the moment to assess and monitor ACHD with a single parameter or a simple scale that would apply to the whole population like the NYHA classification in heart failure patients. Patients with CHD are heterogeneous population and personalized assessment of patients' functional state is important not only for their diagnosis and management, but also for prognostic reasons. Considering all available diagnostic tools, selected CPET parameters, echocardiography and magnetic resonance imaging as well as NT-proBNP and GDF15 levels are valuable assessment tools of ACHD patients which provide the most information about patients and their prognosis. The most promising CPET parameters seem to be peak VO_2_, VE/VCO_2_, heart rate reserve. Due to the heterogeneity of this group of patients, relatively small amount of medical centers dealing with congenital heart diseases, lack of large multi-center studies and uneven access of medical centers to all presented factors, it is a very difficult task to create uniform rules for managing and monitoring the population of CHD patients.

## Author Contributions

PL, MB, MM, and AB-D contributed to conception and design of the paper. PL and AB-D managed the literature research. PL wrote the first draft of the manuscript. MB, MM, and AB-D reviewed and made corrections to the manuscript. All authors contributed to manuscript final revision, read, and approved the submitted version.

## Funding

This project was financed by the Polish Mother's Memorial Hospital Research Institute.

## Conflict of Interest

The authors declare that the research was conducted in the absence of any commercial or financial relationships that could be construed as a potential conflict of interest.

## Publisher's Note

All claims expressed in this article are solely those of the authors and do not necessarily represent those of their affiliated organizations, or those of the publisher, the editors and the reviewers. Any product that may be evaluated in this article, or claim that may be made by its manufacturer, is not guaranteed or endorsed by the publisher.
